# The Accurate Interpretation and Clinical Significance of Morphological Features of Fine Needle Aspiration Cells in Papillary Thyroid Carcinoma

**DOI:** 10.1155/2023/9397755

**Published:** 2023-05-03

**Authors:** Xue-Jiao Xiong, Ming-Ming Xiao, Yi-Xia Zhang, Dong-Ge Liu, Mu-Lan Jin, Jian Wang, Hong-Tao Xu, Qing-Chang Li, Guang-Ping Wu

**Affiliations:** ^1^Department of Pathology, The First Hospital of China Medical University, Shenyang 110001, China; ^2^Department of Pathology, The People's Hospital of Liaoning Province, Shenyang 110016, China; ^3^Department of Ultrasound, The First Hospital of China Medical University, Shenyang 110001, China; ^4^Department of Pathology, Beijing Hospital, National Geriatric Center, Beijing 100730, China; ^5^Department of Pathology, Beijing Chao-Yang Hospital, Capital Medical University, Beijing 100020, China

## Abstract

Papillary thyroid carcinoma (PTC) is the most common malignant neoplasm of the thyroid gland; fine needle aspiration cytology is the most basic and reliable diagnostic method before PTC operation. However, it is not clear which cell morphological changes can be used as a reliable standard for the diagnosis of PTC. A retrospective analysis was performed on 337 patients with PTC confirmed by postoperative histology. An additional 197 randomly selected patients with benign thyroid lesions were included in the study and used as a control group. True papillary arrangements, swirl arrangements, and escape arrangements had high specificity, all of which were 100%, but only swirl arrangements had ideal sensitivity (77.61%). The nuclear volume characteristics had a high sensitivity of more than 90%, but the specificities of both nuclear crowding and nuclear overlap were too low, only 16.34% and 23.35%. The sensitivities of five nuclear structural characteristics were more than 90%, but only the specificity of intranuclear cytoplasmic pseudoinclusions (INCIs) reached 100%, nuclear contour irregularity and pale nuclei with powdery chromatin also had ideal interpretation value except for grooves and marginally placed micronucleoli. Although the sensitivity of psammoma bodies (PBs) was low, the specificity was 100%. In terms of preparation methods, the method of liquid-based preparation (LBP) is obviously better than that of conventional smears. The diagnostic efficiency by the combined detection method of parallel tests showed that without reducing the specificity, the sensitivity increased with the increase of the number of morphological characteristics and finally reached 98.81%. The INCIs and swirl arrangements are the most common and important indicators for the diagnosis of PTC, whereas papillary-like arrangements, the crowding and overlap of nuclear, grooves, marginally placed micronucleoli, and multinucleated giant cells are of little significance for the diagnosis of PTC.

## 1. Introduction

Papillary thyroid carcinoma (PTC) is the most common malignant neoplasm of the thyroid gland, accounting for approximately 90% of all thyroid malignant tumors [1]. A large number of epidemiology confirmed that the incidence of PTC has increased in recent years [2, 3]. Due to the abundant blood vessels in thyroid tissue, histological biopsy cannot be taken in order to avoid massive bleeding. Therefore, fine needle aspiration (FNA) cytology is the most basic and reliable preoperative diagnostic method [4, 5]. In recent years, with the rapid development of ultrasound imaging, the tiny nodules in the thyroid are clearly displayed, which not only provides a prerequisite for FNA but also shows a new challenge to the cytological diagnosis of PTC [6]. FNA is a minimally invasive method, which is suitable for almost all patients with thyroid diseases. There will be no postoperative complications, so the patients themselves are easy to accept. Although BRAF(V600E) detection can provide a reliable basis for mutation, the sensitivity is not satisfactory [7]. Because there are many detection methods, sometimes the results are inconsistent [8]; therefore, BRAF(V600E) detection can only be used as an auxiliary diagnostic method of cytology but cannot completely replace cytology.

Although some cell arrangements and morphological characteristics of PTC are described in the second edition of thyroid the Bethesda System (TBS) reporting system, these features still need to be further revised and improved to meet the needs of clinical cytological interpretation of PTC [9, 10]. The cell arrangements and morphological characteristics of PTC, very like the prognosis of PTC, are very close to that of benign tumors, thus, difficult to distinguish from benign thyroid lesions. Therefore, the accurate cytological diagnosis of PTC still needs in-depth research and discussion to further refine its diagnostic criteria. The main purpose of this study was to evaluate the cell arrangement characteristics, nuclear volume characteristics, and nuclear structural characteristics of PTC by FNA cytology. In particular, we aimed to evaluate the diagnostic efficiency of combined detecting changes of 100% specificity.

## 2. Materials and Methods

This study was a retrospective analysis of cytological diagnoses in patients with PTC patients confirmed by postoperative histology. The study was approved by the Ethics Committee of the First Hospital of China Medical University, and informed consent was obtained from each patient. The study also included the pathological diagnosis of both histology and cytology from the Department of Pathology at the First Hospital of China Medical University during the period March 2019–August 2021. A total of 337 patients with PTC were recruited in this study, including 91 cases of classic PTC and 246 cases of micro-PTC. The study group involved with 71 men and 266 women, ranging in age from 21 to 71 years. An additional 197 randomly selected patients with benign thyroid lesions were included in the study and used as a control group, including 71 cases of thyroid adenoma, 64 cases of nodular goiter, 23 cases of Hashimoto's disease, and 39 cases of thyroid adenoma complicated with nodular goiter.

All FNA procedures were performed by two experienced puncture doctors using the mindray Eagle R9 (mindray, Shenzhen, China) color Doppler ultrasound system, using a linear array probe at a frequency of 3–11 MHz. The puncture needle was a 23G needle (Gallini S.R.L., Mantova, Italy). Each thyroid nodule was punctured three times.

The cells obtained for the first time performed the conventional smears for two slides, fixed in 95% alcohol, and stained by Papanicolaou's method. The remaining cells were rinsed into the small vials containing SurePath preservative fluid (BD Tripath, Burlington, NC, USA) for the liquid-based Pap test (LBP). The cells obtained for the second time were rinsed into 1.5 ml eppendorf (EP) tubes containing AmoyDx preservative fluid (AmoyDx, Xiamen, China) for BRAF gene detection on an ABI 7500 genetic analyzer (Applied Biosystems, CA, USA). The cells obtained for the third time were also rinsed into the small vials containing the remaining cells of the first time for the automatically prepared and stained of slides by AutoCyte PREP system (BD Tripath, Burlington, NC, USA). In this way, the small vials actually contain the cell components obtained for the first and third times. Both conventional smears and LBP slides were interpreted and reported by experienced cytopathologists, and the findings were recorded into smear and LBP groups, respectively.

Statistical analysis was performed using SPSS 16.0 software package (SPSS, Inc. Chicago, IL, USA). The chi-square test or Fisher's exact test was used for the comparison of positive cases between different groups. A *P*-value of less than 0.05 was considered statistically significant.

## 3. Results

First of all, by comparing the clinical characteristics of PTC and benign lesions, we found that the onset age of PTC was mainly under 40 years old, and the onset ages of both classic PTC and micro-PTC were significantly lower than that of benign lesions. However, there was no correlation with sex. The size and definition of thyroid nodules were consistent. The micro-PTC was mainly concentrated in 0.3–0.9 cm, and the classic PTC was concentrated in 1–4.5 cm. The results are shown in Table 1.

**Figure 1:**
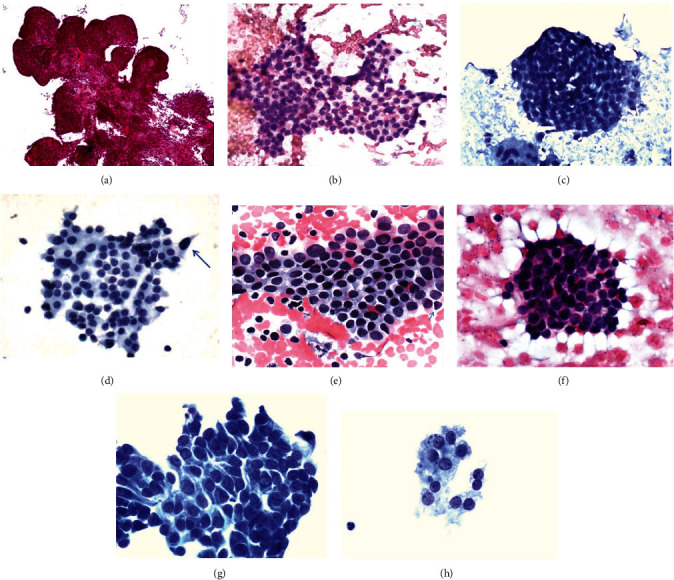
The morphological features of fine needle aspiration cells in PTC (Papanicolaou stain). (a) True papillary arrangements. The comprised of fibrovascular cores lined by neoplastic cells are seen in the conventional type of papillary thyroid carcinoma (smear, ×100). (b) Papillary-like arrangements. The cells are arranged into multi-level finger protrusions, there is no fibrovascular core in the protrusion, and the surface of the protrusion is smooth (smear, ×400). (c) Swirl arrangements. The figure shows a concentric aggregate composed of more than 100 tumor cells, the most peripheral cells are oval, and the long axis of the cells is perpendicular to the radius of the vortex. At the lower left corner of the vortex arrangement is a multinucleated giant cell (smear, ×400). (d) Escape arrangements. The arrow in the figure shows that a cell is separated from the original cell cluster, and there is obvious atypia at the edge of the follicular cell cluster. Its cytoplasm may still be partially continuous with the cell cluster (LBP, ×400). (e) The enlargement, elongation, contour irregularity, and molding of nuclear. The area or length of some nuclei is significantly more than 1.5 times that of same kind cells, several cells in the upper left corner show obvious nuclear contour irregularity, the rightmost arrow shows that the nucleus shows local molding (smear, ×400). (f) The crowding and overlap of nuclear. The distance between adjacent cells is significantly reduced, and even one nucleus covers another nucleus (smear, ×400). (g) INCIs and nuclear grooves are shown. Note that the two INCIs share the same aqua color and granular texture as the surrounding cytoplasm and that several peripheral cells show longitudinal nuclear sulcus (LBP, ×400). (h) Pale nuclei and nucleoli are shown. Close inspection at high magnification shows pale nuclei with powdery chromatin and micronucleoli edge set (LBP, ×400).

Second, we need to interpret the common cell arrangements of PTC and compare them with those of benign thyroid lesions, so as to make up for the lack of tissue structure in cytology. The present study found that the common cell arrangements include the following four types. True papillary arrangements (Figure 1(a)): the cells are arranged in papillary shape, the papilla is lined with a fibrovascular core, and the surface of the papilla is covered with dwarf columnar cells with obvious atypia; papillary-like arrangements (Figure 1(b)): the cells are arranged into multi-level finger protrusions, there is no fibrovascular core in the protrusion, and the surface of the protrusion is smooth; swirl arrangements (Figure 1(c)): it is a concentric aggregate composed of about 50–200 tumor cells, the most peripheral cells are oval, and the long axis of the cells is perpendicular to the radius of the vortex; escape arrangements (Figure 1(d)): one or a few cells separated from the original cell cluster and with obvious atypia appear at the edge of the follicular cell cluster, and their cytoplasm may still be partially continuous with the cell cluster. The present study showed that true papillary arrangements, swirl arrangements, and escape arrangements had high specificity, all of which were 100%, but only swirl arrangements had ideal sensitivity (77.74%), which was significantly better than true papillary arrangements and escape arrangements (*P* < 0.01). The results are shown in Table 2.

The nuclear volume characteristics of PTC are usually shown as nuclear enlargement, nuclear elongation, nuclear crowding, nuclear overlap, and nuclear molding. Nuclear enlargement (Figure 1(e)) is usually defined as that the area of the nucleus exceeds more than 1.5 times that of the benign nucleus of the same kind, and nuclear elongation (Figure 1(e)) refers to that the length exceeds more than 1.5 times when the width of the nucleus remains unchanged. When the distance between adjacent cells is significantly reduced, it is called nuclear crowding (Figure 1(f)). When one nucleus covers another nucleus, it is called nuclear overlap (Figure 1(f)), and an enlarged nucleus causes the deformation of adjacent nuclei, which is called nuclear molding (Figure 1(e)). This study found that the above nuclear volume characteristics showed a high sensitivity of more than 90%, but the specificities of both nuclear crowding and nuclear overlap were too low, significantly lower than those of the other three nuclear volume characteristics (*P* < 0.01). The results are shown in Table 3.

The nuclear structural characteristics of thyroid lesions are not only an important clue for cytological diagnoses but also the key point to distinguish benign and malignant lesions. This study found that the five nuclear structural characteristics all had more than 90% sensitivity. However, only had the specificity of intranuclear cytoplasmic pseudoinclusions (INCIs) (Figure 1(g)) reached 100%, nuclear contour irregularity (nuclear CI) (Figure 1(e)) and pale nuclei with powdery chromatin (pale nuclei) (Figure 1(h)) also had ideal interpretation value, but the specificities of both longitudinal nuclear grooves (grooves) (Figure 1(g)) and marginally placed micronucleoli (nucleoli) (Figure 1(h)) were too low, which were significantly lower than that of pale nuclei (*P* < 0.05 or 0.01). The results are shown in Table 4.

In addition, we studied the smear or slide background, preparation method, and gene detection results of patients with PTC and benign lesions in detail. The results showed that although the sensitivity of psammoma bodies (PBs) was low, the specificity was 100%. In terms of preparation methods, LBP is obviously better than conventional smear method (*P* < 0.05). When the specificity of BRAF gene detection is 100%, the sensitivity is also ideal (78.51%). The results are shown in Table 5.

Finally, the morphological characteristics of PTC with 100% specificity were gathered, and the diagnostic efficiency was evaluated by the combined detection method of parallel tests. The results showed that without reducing the specificity, the sensitivity increased with the increase of the number of morphological characteristics, and finally reached 98.81%. However, there was little difference in sensitivity between groups, *p* > 0.05. The results are shown in Table 6.

## 4. Discussion

The thyroid TBS reporting system mentioned three common cell arrangements of PTC, namely true papillary fragments, papillary-like fragments, and cellular swirls [11, 12]. However, the sensitivity and specificity of these three arrangements for the diagnosis of PTC were not described in detail, and which of the three arrangements were more reliable for the interpretation of PTC was not compared either. The word fragment is seen macroscopically, not the microscopic form observed under the microscope. Therefore, we think it is more appropriate to replace the word fragments with the word arrangements. This study found that the true papillary arrangements and swirl arrangements showed high specificities, both of which were 100%, but the sensitivity of true papillary arrangements was too low (21.49%), only the sensitivity of swirl arrangements was ideal (77.61%). Swirl arrangements, sometimes also called an “onion-skin” pattern, are a distinctive feature of the cell arrangements, and benign thyroid nodules have not been reported [13, 14]. In addition to the above three arrangements, we also found a pattern of escape arrangements, which we recently found and reported in the interpretation of lung adenocarcinoma cells [15]. The escape arrangements of PTC may be characterized by the loss of cellular polarity and cohesiveness. Although the sensitivity of escape arrangements is low, the specificity is 100%. According to the above arrangements, especially the arrangements with 100% specificity, it is suggested that when interpreting the morphological characteristics of PTC, we should pay attention to observing the arrangements of cells, which have important clinical application value for the diagnosis of PTC. Although the smears or LBP cannot observe the tissue structure, the arrangements of cells can indirectly reflect some characteristics of tissue structure, which are not available in benign lesions.

The nuclear volume characteristics of PCT are not only important clues for cytological diagnoses but also the focus of cytopathologists. However, since there are many parameters related to nuclear volume characteristics, it is unclear whether these parameters are equally important for the diagnosis of PTC. The five nuclear volume characteristics included in this study had more than 90% high sensitivity, but the specificity was not high, especially the specificity of both nuclear crowding and nuclear overlap was too low, respectively 16.34% and 23.35%, which means that nuclear crowding and nuclear overlap are also common in benign lesions, so they are not helpful for the diagnosis of PTC. From another point of view, it can be found that the prognosis of PTC is very good, which may be closely related to the relatively small nuclear atypia. These results suggest that it is not enough to rely only on the both crowding and overlap of nuclear in the diagnosis of PTC. We must deeply explore other cell morphological features of PTC in order to meet the clinical needs.

The nuclear structural characteristics of PTC are also the focus of cytopathologists, but which can be used as a reliable parameter to interpret PTC is not clear. This study found that nuclear CI, grooves, INCIs, pale nuclei, and nucleoli all showed a high sensitivity of more than 90%, but only INCIs showed a very ideal specificity of 100%, i.e., INCIs were not seen in benign thyroid nodules. This result is not completely consistent with the results described in TBS for Reporting Thyroid Cytopathology [11, 12]. TBS for Reporting Thyroid Cytopathology states that in very rare cases, INCIs can also be seen in benign thyroid nodules and lymphocytic thyroiditis. A true INCI displays the same color/texture of adjacent cytoplasm and is sharply bordered by a rim of condensed chromatin, like a “wire loop.” These features help distinguish INCIs from common mimics: degenerative and artifactual vacuoles, fixation artifacts, and superimposed red blood cells. Therefore, we believe that if INCIs are found in benign thyroid lesions, it must be carefully observed and checked according to the criteria in the definition to avoid false positive results. Suzuki et al. reported that the specificity of bare nucleoli is as high as 96.1%, which may become a new indicator of PTC in LBP [16]. However, this study found that only 33 of 337 cases of PTC showed bare nucleoli, with a sensitivity of 9.79%, and 5 of 197 cases of benign lesions also found bare nucleoli. Rupp and Ehya reported that a large number of visible nuclear grooves can be considered as a reliable criterion for the diagnosis of PTC [17]. However, it is no accident that this study found that more nuclear grooves can also be seen in nearly half of benign lesions. Due to the limited number of cases in this study, the accurate interpretation significance of both bare nucleoli and nuclear grooves cannot be determined, which is also the limitation of this study. It is necessary to further expand the number of cases in future studies to explore the accurate interpretation significance of these two features.

This study also found that PBs in the slide background showed important diagnostic value for the interpretation of PTC. Although the sensitivity of PBs is low, its specificity is 100%. However, the result is not consistent with the conclusions reported in the literature. Ellison et al. believe that isolated PBs are an unreliable predictor of PTC in the absence of cytologic features [18]. The value of accurate interpretation needs to be further discussed by expanding the sample size in the future. When the two preparation methods of LBP slides and conventional smears are compared, it was found that the cytological features of slides are more common than smears, but LBP could not completely replace the smears. In 81 cases of PTC, slides showed negative results, but typical cancer cells were found in the smears. Similarly, in 212 slide positive cases, smears showed negative results. Therefore, we advocate that LBP slides and conventional smears should be applied together, combined and complementary to each other. In addition, we evaluated the gene detection of the same group of cases and found that the sensitivity was 78.34%, which was slightly higher than that reported (76.71%) in the literature [19]. At the same time, it also suggests that gene detection cannot solve all PTC cases. A preoperative FNA cytology diagnosis is an indispensable clinical diagnostic method.

In order to maximize the diagnostic efficiency, we conducted combined detection of morphological features with 100% specificity. The results showed that the sensitivity reached 98.81% without reducing the specificity. It has been reported that a definitive diagnosis of PTC should be reserved for cases that have, in addition to other characteristic features, at least one of the following: true papillary arrangements, PBs, and INCIs [20–22]. This study found that in addition to the true papillary arrangements, PBs, and INCIs, there were swirl arrangements and escape arrangements, both the specificities of them are also 100%. Therefore, we believe that swirl arrangements and escape arrangements are equally important with the above three morphological features, even INCIs and swirl arrangements are the most common and important indicators for the diagnosis of PTC. When interpreting the morphological features of follicular cells, if one of the above five morphological features is found, it should be diagnosed as suspicious PTC. When more than two of the above five features are found, it should be determined as the diagnosis of PTC.

## 5. Conclusion

True papillary arrangements, swirl arrangements, escape arrangements, INCIs, and PBs have 100% specificity, and the escape arrangements of PTC are the first discovery in this study. When using a single detection, the sensitivity of INCIs is the highest (97.01%), while when using parallel test for combined detection, the sensitivity can be as high as 98.81%, and the specificity is always 100%. The characteristics of cell arrangements, nuclear volume, nuclear structure, and slide or smear background are very important for the interpretation of FNA cells in patients with PTC, but papillary-like arrangements, the crowding and overlap of nuclear, grooves, marginally placed micronucleoli, and multinucleated giant cells are of little significance for the diagnosis of PTC.

## Figures and Tables

**Table 1:** The clinical characteristics of patients with papillary thyroid carcinoma and benign lesions (%).

Groups	*n*	Age		Gender		Nodule size	
		21–40	41–71	Male	Female	0.3–0.9 cm	1–4.5 cm
PTC	337	139∗∗	198	71	266	246	91
Classic PTC	91	38∗	53	21	70	0	91∗∗
Micro-PTC	246	101∗	145	50	196	246∗∗	0
BL	197	50	147	38	159	128	69

PTC: papillary thyroid carcinoma; BL: benign lesions.

∗*P* < 0.05 as compared to benign, ∗∗*P* < 0.01 as compared to benign.

**Table 2:** The cell arrangement characteristics of patients with papillary thyroid carcinoma and benign lesions (%).

Groups	*n*	True papillae	Papillae-like	Swirl	Escape
PTC	337	70 (20.77)	222 (65.88)	262 (77.74)	85 (25.22)
BL	197	0 (0.00)	48 (24.37)	0 (0.00)	0 (0.00)
TAD	71	0 (0.00)	27 (38.03)	0 (0.00)	0 (0.00)
NG	64	0 (0.00)	8 (12.5)	0 (0.00)	0 (0.00)
HD	23	0 (0.00)	2 (8.70)	0 (0.00)	0 (0.00)
NG and TAD	39	0 (0.00)	11 (28.21)	0 (0.00)	0 (0.00)
Sensitivity	(95% CI)	20.77 (±4.33)	65.88 (±5.06)	77.74 (±4.44)∗∗	25.22 (±4.64)
Specificity	(95% CI)	100.00 (±0)	75.63 (±5.99)	100.00 (±0)	100.00 (±0)
Accuracy	(95% CI)	50.00 (±4.24)	69.48 (±3.91)	85.96 (±2.95)	52.81 (±4.23)

PTC: papillary thyroid carcinoma; BL: benign lesions; TAD: thyroid adenoma; NG: nodular goiter; HD: Hashimoto's disease.

∗∗*P* < 0.01 as compared to true papillae or escape.

**Table 3:** The nuclear volume characteristics of patients with papillary thyroid carcinoma and benign lesions (%).

Groups	*n*	Enlargement	Elongation	Crowding	Overlap	Molding
PTC	337	249 (73.89)	333 (98.81)	332 (98.52)	332 (98.52)	307 (91.10)
BL	197	40 (20.30)	55 (27.92)	165 (83.76)	151 (76.65)	29 (14.72)
TAD	71	20 (28.17)	31 (43.66)	63 (88.73)	63 (88.73)	11 (15.49)
NG	64	4 (6.25)	12 (18.75)	47 (73.44)	40 (62.5)	7 (10.94)
HD	23	4 (17.39)	6 (26.09)	23 (100.00)	20 (86.96)	8 (34.78)
NG and TAD	39	12 (30.77)	6 (15.38)	32 (82.05)	28 (71.79)	3 (7.69)
Sensitivity	(±95% CI)	73.89 (±4.69)	98.81 (±1.16)	98.51 (±1.29)	98.51 (±1.29)	91.10 (±3.04)
Specificity	(±95% CI)	79.70 (±5.62)∗∗	72.08 (±6.26)∗∗	16.24 (±5.15)	23.35 (±5.91)	85.28 (±4.95)∗∗
Accuracy	(±95% CI)	76.03 (±3.62)	88.95 (±2.66)	68.16 (±3.95)	70.79 (±3.86)	88.95 (±2.66)

PTC: papillary thyroid carcinoma; BL: benign lesions; TAD: thyroid adenoma; NG: nodular goiter; HD: Hashimoto's disease.

∗∗*P* < 0.01 as compared to crowding or overlap.

**Table 4:** The nuclear structural characteristics of patients with papillary thyroid carcinoma and benign lesions (%).

Groups	*n*	Nuclear CI	Grooves	INCIs	Pale nuclei	Nucleoli
PTC	337	333 (98.81)	321 (95.25)	328 (97.32)	333 (98.81)	309 (91.69)
BL	197	35 (17.77)	96 (48.73)	0 (0.00)	54 (27.415)	105 (53.30%)
TAD	71	19 (26.76)	44 (61.97)	0 (0.00)	27 (38.03)	42 (59.15)
NG	64	8 (12.5)	20 (31.25)	0 (0.00)	4 (6.25)	28 (43.75)
HD	23	4 (17.39)	12 (57.14)	0 (0.00)	12 (57.14)	21 (91.30)
NG and TAD	39	4 (10.26)	20 (51.28)	0 (0.00)	11 (28.21)	14 (35.90)
Sensitivity	(±95% CI)	98.81 (±1.16)	95.25 (±2.27)	97.33 (±1.72)	98.81 (±1.16)	91.69 (±2.95)
Specificity	(±95% CI)	82.23 (±5.34)	51.27 (±6.98)∗	100.00 (±0)	72.59 (±6.23)	46.70 (±6.97)^**^
Accuracy	(±95% CI)	92.70 (±2.21)	79.02 (±3.45)	98.31 (±1.09)	89.14 (±2.64)	75.09 (±3.67)

PTC: papillary thyroid carcinoma; BL: benign lesions; TAD: thyroid adenoma; NG: nodular goiter; HD: Hashimoto's disease; nuclear CI: nuclear contour irregularity; grooves: longitudinal nuclear grooves; INCIs: intranuclear cytoplasmic pseudoinclusions; pale nuclei: pale nuclei with powdery chromatin; nucleoli: marginally placed micronucleoli.

∗*P* < 0.05 as compared to pale nuclei, ∗∗*P* < 0.01 as compared to pale nuclei.

**Table 5:** The results of smear background, preparation method, and gene detection of patients with papillary thyroid carcinoma and benign lesions (%).

Groups	*n*	PBs	MGCs	Smears	LBP	BRAF
PTC	337	81 (24.04)	107 (31.75)	125 (37.09)	256 (75.96)	264 (78.34)
BL	197	0 (0.00)	90 (45.69)	18 (9.14)	179 (90.86)	0 (0.00)
TAD	71	0 (0.00)	41 (57.75)	4 (5.63)	67 (94.37)	0 (0.00)
NG	64	0 (0.00)	24 (37.5)	2 (3.13)	62 (96.88)	0 (0.00)
HD	23	0 (0.00)	11 (47.83)	6 (26.09)	17 (73.91)	0 (0.00)
NG and TAD	39	0 (0.00)	14 (35.90)	6 (15.38)	33 (84.62)	0 (0.00)
Sensitivity %	(±95% CI)	24.04 (±4.56)	31.75 (±4.97)	37.09 (±5.16)	75.96 (±4.56)∗	78.34 (±4.40)
Specificity %	(±95% CI)	100.00 (±0)	54.31 (±6.96)	90.86 (±4.02)	9.14 (±4.02)	100.00 (±0)
Accuracy %	(±95% CI)	52.06 (±4.23)	40.07 (±4.16)	56.93 (±4.20)	51.31 (±4.24)	86.33 (±2.91)

PTC: papillary thyroid carcinoma; BL: benign lesions; TAD: thyroid adenoma; NG: nodular goiter; HD: Hashimoto's disease; PBs: psammoma bodies; MGCs: multinucleated giant cells; LBP: liquid-based preparation; GD: gene detection; ∗*P* < 0.05 as compared to smears.

**Table 6:** The diagnostic performance of combined interpretation via morphological characteristics of PTC (100%).

Performance	INCIs	+Swirls	+Escape	+PBs	+True papillae
Sensitivity (±95% CI)	97.32 (±1.72)	98.21 (±1.41)	98.81 (±1.15)	98.81 (±1.15)	98.81 (±1.15)
Specificity (±95% CI)	100 (±0)	100 (±0)	100 (±0)	100 (±0)	100 (±0)
PPV (±95% CI)	100 (±0)	100 (±0)	100 (±0)	100 (±0)	100 (±0)
NPV (±95% CI)	95.63 (±2.79)	97.04 (±2.32)	98.01 (±1.93)	98.01 (±1.93)	98.01 (±1.93)
Accuracy (±95% CI)	98.31 (±1.09)	98.87 (±0.89)	99.25 (±0.73)	99.25 (±0.73)	99.25 (±0.73)

INCIs: intranuclear cytoplasmic pseudoinclusions; PBs: psammoma bodies; NPV: negative predictive value; PPV: positive predictive value.

## Data Availability

All data generated or analyzed during this study are included in this article. Further enquiries can be directed to the corresponding author.
